# Genome Sequences of *Klebsiella variicola* Isolates from Dairy Animals with Bovine Mastitis from Newfoundland, Canada

**DOI:** 10.1128/genomeA.00938-15

**Published:** 2015-09-10

**Authors:** Fraser W. Davidson, Hugh G. Whitney, Kapil Tahlan

**Affiliations:** Department of Biology, Memorial University of Newfoundland, St. John’s, Newfoundland and Labrador, Canada

## Abstract

*Klebsiella variicola* was recently reported as an emerging and/or previously misidentified species associated with opportunistic infections in humans. Here, we report the draft genome sequences of *K. variicola* isolates from two animals with clinical mastitis from a dairy farm in Newfoundland, Canada.

## GENOME ANNOUNCEMENT

The genus *Klebsiella* comprises species capable of colonizing diverse environmental niches (1) and includes the opportunistic human pathogens *K. pneumoniae* and *K. oxytoca* (2, 3). In comparison, *Klebsiella variicola* was first reported as a nonpathogenic plant-associated species (2), which was also isolated from the nests of leafcutter ants (strain AT-22) and from sugarcane (strain DX120E) (4, 5). Recently, pathogenic strains of *K. variicola* (Bz19 and 8917) were also reported in humans (3, 6), with the species being associated with higher rates of mortality in patients with bloodstream infections than those infected with *K. pneumoniae* (7). In a previous study, *K. variicola* was isolated from dairy animals with clinical mastitis (isolates NL49 and NL58 from the island of Newfoundland, Canada) (8), a condition normally associated with other opportunistic pathogenic *Klebsiella* species from the environment. Therefore, the genomes of *K. variicola* NL49 and NL58 were sequenced to identify potential virulence determinants and for source-tracking purposes.

*K. variicola* NL49 and NL58 were cultured as described previously (8), and genomic DNA was isolated using the QIAamp DNA minikit (Qiagen Sciences, USA). Nextera XT libraries were prepared, and sequence data were obtained using the Illumina HiSeq platform (Illumina, Inc., USA) at the Centre for Applied Genomics, The Hospital for Sick Children, Toronto, Canada. *De novo* assembly of the two genomes was carried out using the A5 pipeline (9). Annotations were performed using the NCBI Prokaryotic Genome Annotation Pipeline and the RAST servers (10–12), which were further analyzed using the Geneious R8 software package (Biomatters Ltd., New Zealand).

In total, 147 and 145 contigs were obtained for NL49 and NL58, respectively. The two genomes were found to be identical (approximately 5.7 Mb each), even though the samples originated from two different animals from the same farm (8), suggesting that a single *K. variicola* strain was associated with the infection. The draft genomes contain sequences for a conjugative plasmid (NL49 scaffold 34 and NL58 scaffold 36), genes for nitrogen fixation, and genes for type II and type VI secretion systems. NL49/58 have typical sequences for *K. variicola* housekeeping genes, whereas the genes involved in capsule biosynthesis show varied degrees of similarity with that reported in *K. pneumoniae*. The inability of *K. variicola* to ferment adonitol is used as a test to differentiate it from *K. pneumoniae*, which has been questioned in the past (13). Previously, NL49/58 tested positive for adonitol fermentation (8), but the characteristic genes involved in the process (14) were not detected in the draft genome sequences. Therefore, it is possible that some *K. variicola* strains ferment adonitol using a different/modified pathway compared to that with *K. pneumoniae*. In summary, the genome sequences of *K. variicola* NL49/NL58 will be used to explore the evolution and emergence of this species as an animal/human pathogen in the future.

### Nucleotide sequence accession numbers. 

The whole-genome shotgun projects for the two *K. variicola* isolates have been deposited in GenBank under the accession numbers LFYK00000000 (NL49) and LGAW00000000 (NL58). The versions described in this paper are LFYK01000000 and LGAW01000000, respectively.
